# Association between the United Arab Emirates child safety seat and mandatory seatbelt legislation and child passenger injuries and fatalities

**DOI:** 10.3389/fpubh.2026.1846288

**Published:** 2026-07-20

**Authors:** Muhammad Uba Abdulazeez, Aminu S. Abdullahi, Sjaan Koppel, David Alao, Kassim Abdullah

**Affiliations:** 1 Emirates Centre for Mobility Research, United Arab Emirates University, Al Ain, United Arab Emirates; 2 Department of Mechanical & Aerospace Engineering, College of Engineering, United Arab Emirates University, Al Ain, United Arab Emirates; 3 Department of Automotive Engineering, Faculty of Engineering and Engineering Technology, Abubakar Tafawa Balewa University, Bauchi, Nigeria; 4 Institute of Public Health, College of Medicine and Health Sciences, United Arab Emirates University, Al Ain, United Arab Emirates; 5 Monash University Accident Research Centre, Monash University, Melbourne, VIC, Australia; 6 Department of Internal Medicine, United Arab Emirates University, Al Ain, United Arab Emirates

**Keywords:** child occupant, child safety seat, injury severity, legislation, seatbelt, UAE

## Abstract

Child occupants in the United Arab Emirates (UAE) have one of the highest crash-related injury and fatality rates globally. The UAE child passenger legislation (CPL) enacted in 2017 mandates the use of child safety seats for child occupants until age of 4 years and seatbelt use from age of 5. The current study aimed to evaluate this legislation’s association with reductions in crash-related injuries and trauma (Injury Severity Score [ISS]) among child occupants aged 0–14 years. Additionally, the study examined whether the relationship between crash injury characteristics and injury severity changed after the implementation of the CPL. The child occupant injury data was obtained from the UAE Ministry of Interior for 5 years (2015–2019), while the trauma data was obtained from Al Ain Hospital for 6 years (2014–2020). Poisson regression analyses were used to compare the pre-CPL and post-CPL periods. The CPL was significantly associated with 62 and 85% reductions in the crude (IRR: 0.38, CI: 0.16–0.81) and adjusted (IRR: 0.15, CI: 0.01–0.83) rates of fatal injuries per 1000 crashes among children aged 0–4 years. Equally, the CPL was significantly associated with a 66% reduction in the crude rate of fatal injuries (IRR: 0.34, CI: 0.10–0.95) per 1000 crashes for children aged 5–9 years. Conversely, the CPL was significantly associated with a 75% increase in the adjusted rate of minor injuries (IRR: 1.75, CI: 1.06–2.95) per 1000 crashes for children aged 10–14 years. These findings will provide key insights for policymakers and road safety practitioners, thereby informing both legislative and non-legislative interventions aimed at strengthening child occupant protection and ensuring children travel while they are safely and securely restrained.

## 1 Introduction

Road traffic crashes (RTCs) are the leading cause of injuries and deaths for children aged 0–14 years in the United Arab Emirates (UAE) (1). Child vehicle occupants represent the majority of children injured due to RTCs in the UAE (2). Several child occupant safety initiatives have been launched in the UAE since 2010. These include promotional activities, media outreach, training programs, awareness initiatives, and educational campaigns. Additionally, free child safety seats (CSS) have been distributed to thousands of families with a child or those expecting a baby (3, 4).

In 2017, child passenger legislation (CPL) was introduced in the UAE, mandating the use of CSS for children aged 0–4 years and seatbelts for children aged 5 and older to improve child occupant safety (5). However, standard vehicle restraints are designed for adults and do not adequately protect young passengers (6). The current UAE CPL does not require the use of a CSS after age 5, leaving many children at risk. The vehicle seatbelt is designed to fit the seated height of a 50th percentile adult male (88.4 cm), which is achieved by children between the ages of 15 and 16 years (7). A recent study showed that the majority of children aged 5–12 in the UAE do not meet the seatbelt fit requirements (8).

Therefore, this study was undertaken to evaluate the association between this legislation and reducing crash-related injuries and fatalities, as well as trauma severity for child vehicle occupants aged 0–14 years. This will provide key insights for policymakers and road safety practitioners, thereby informing both legislative and non-legislative interventions aimed at strengthening child occupant protection and ensuring children travel while they are safely and securely restrained.

## 2 Materials and methods

Child RTC data (injury severity) was obtained from the UAE Ministry of Interior (MOI) for 5 years (2015–2019). Additionally, child RTC trauma data (trauma severity) was obtained from Al Ain Hospital for 6 years (2014–2020). Details regarding the variables available in each dataset are provided in the supplementary material (Supplementary Tables A.1, A.2). Additionally, data on the number of crashes, Gross Domestic Product (GDP), the number of driver license holders, and the proportion of young males were obtained from the UAE Federal Competitiveness and Statistics Authority (FCSA). Data for Dubai emirate was not available in a format suitable for analysis and was therefore excluded from the current study. Child vehicle occupants aged 14 years or younger at the time of the crash were selected for inclusion in the present study. The injury severity dataset provides national-level data and was used to evaluate the association between CPL implementation and injury reduction while the trauma dataset was included to complement the national dataset by providing clinical context regarding RTC trauma patterns at the main referral hospital in a major city in the UAE.

The injury severity and trauma datasets were divided into two parts:

- For the injury severity dataset: 2.5 years pre-legislation (January 2015–June 2017) and 2.5 years post-legislation (July 2017–December 2019)and
- For the trauma dataset: 3.5 years pre-legislation (January 2014–June 2017) and 2.75 years post-legislation (July 2017–March 2020).

Injury severity was classified into four levels in the injury severity dataset according to the UAE MOI classification (9, 10):

- Minor: Injuries that required self-care or basic medical intervention.
- Moderate: Injuries that required medical intervention or hospital stay.
- Severe: Injuries that required immediate medical intervention.
- Fatal: Injuries that led to death.and two classes in the trauma dataset
- ISS ≤ 16: minor
- ISS > 16: major

Poisson regression analyses were used to compare the pre-legislation and post-legislation periods. The analyses proceeded in two stages. First, we estimated the effect of the CPL on the absolute counts and crash-standardized rates of child occupant injuries, fatalities, and trauma. Second, we adjusted for covariates to account for potential confounding effects and refine the estimation of the CPL’s impact. These covariates include Gross Domestic Product (GDP), the number of driver license holders (11), and the proportion of young males (12). The outcome variables were the counts and the rates of minor, moderate, severe, and fatal injuries as well as minor and major trauma. The rates were computed per 1000 crashes for the injury dataset and per 100 crashes for the trauma dataset. This rate serves as an indicator of the traffic exposure risk.

As the CPL requires CSS use until the age of 4 and seatbelt use from the age of 5, the analyses were conducted for three age groups as follows:

- 0–4 years: CSS group
- 5–9 years: Seatbelt group 1. We kept this age group separate from the older age group because evidence indicates that CSS better protects this age group than vehicle seatbelt (13–15).
- 10–14 years: Seatbelt group 2: This grouping was based on evidence on the effectiveness of booster seats for this age group compared with the vehicle seatbelt (7, 16).

To assess whether the relationship between crash injury characteristics and injury severity changed after the implementation of the CPL, a binary variable indicating pre and post CPL was created, and interaction terms were developed between this variable and the crash injury characteristics. Variables that showed significant interactions with the binary CPL variable were selected for inclusion in the regression model. Since injury severity is ordinal (minor, moderate, severe, fatal), the proportional odds assumption was checked using the Brant test. While some crash injury characteristics satisfied the proportional odds assumption, others violated it. Accordingly, we fitted a partial proportional odds model whereby variables that satisfied the proportional odds assumption were modelled with constant proportional odds while those that violated it were modelled with partial proportional odds. The potential for multicollinearity among the crash characteristics was assessed using the Variance Inflation Factor (VIF), and all VIFs were <5.

Similarly, binary logistic regression analysis was performed to assess whether the relationship between trauma characteristics and trauma severity changed after the implementation of the CPL. A binary variable indicating pre- and post-CPL was created, and interaction terms were developed between this variable and the trauma characteristics. Variables that showed significant interactions with the binary CPL variable were selected for inclusion in the regression model. The potential for multicollinearity among the trauma characteristics was assessed using the VIF, and all VIFs were <5. Binary regression model diagnostics, including classification accuracy, Area Under the Curve (AUC), and the Likelihood Ratio Test, were conducted. The significance level was set at 0.05, and all analyses were performed using R version 4.5.3. This study was approved by the Al Ain Hospital Research and Ethics Governance Committee (AAHEC-03-20-008).

## 3 Results

### 3.1 Injury and trauma trends

**Figure 1:**
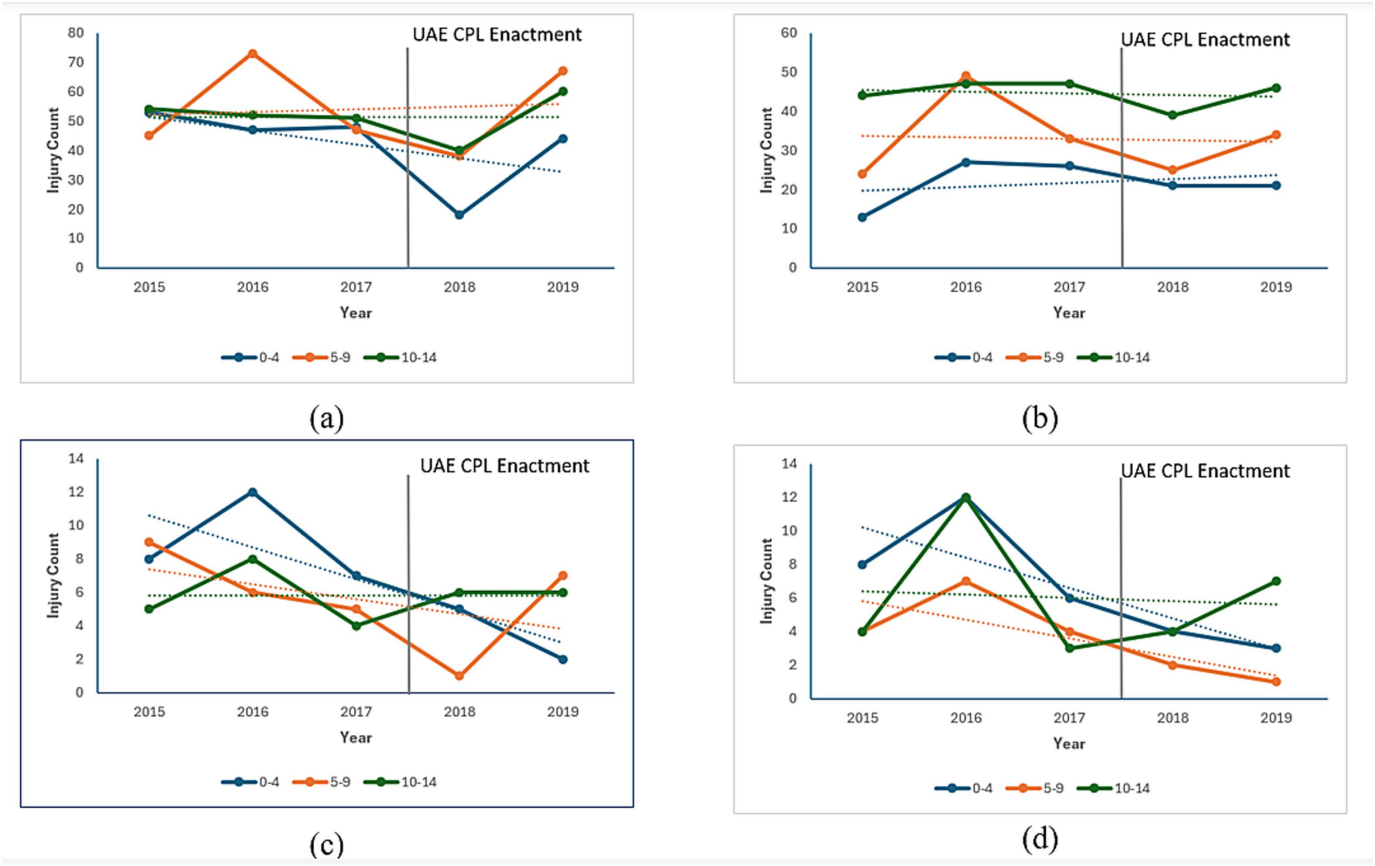
Incidence of child occupant injuries **(a)** minor **(b)** moderate **(c)** severe **(d)** fatal.

**Figure 2:**
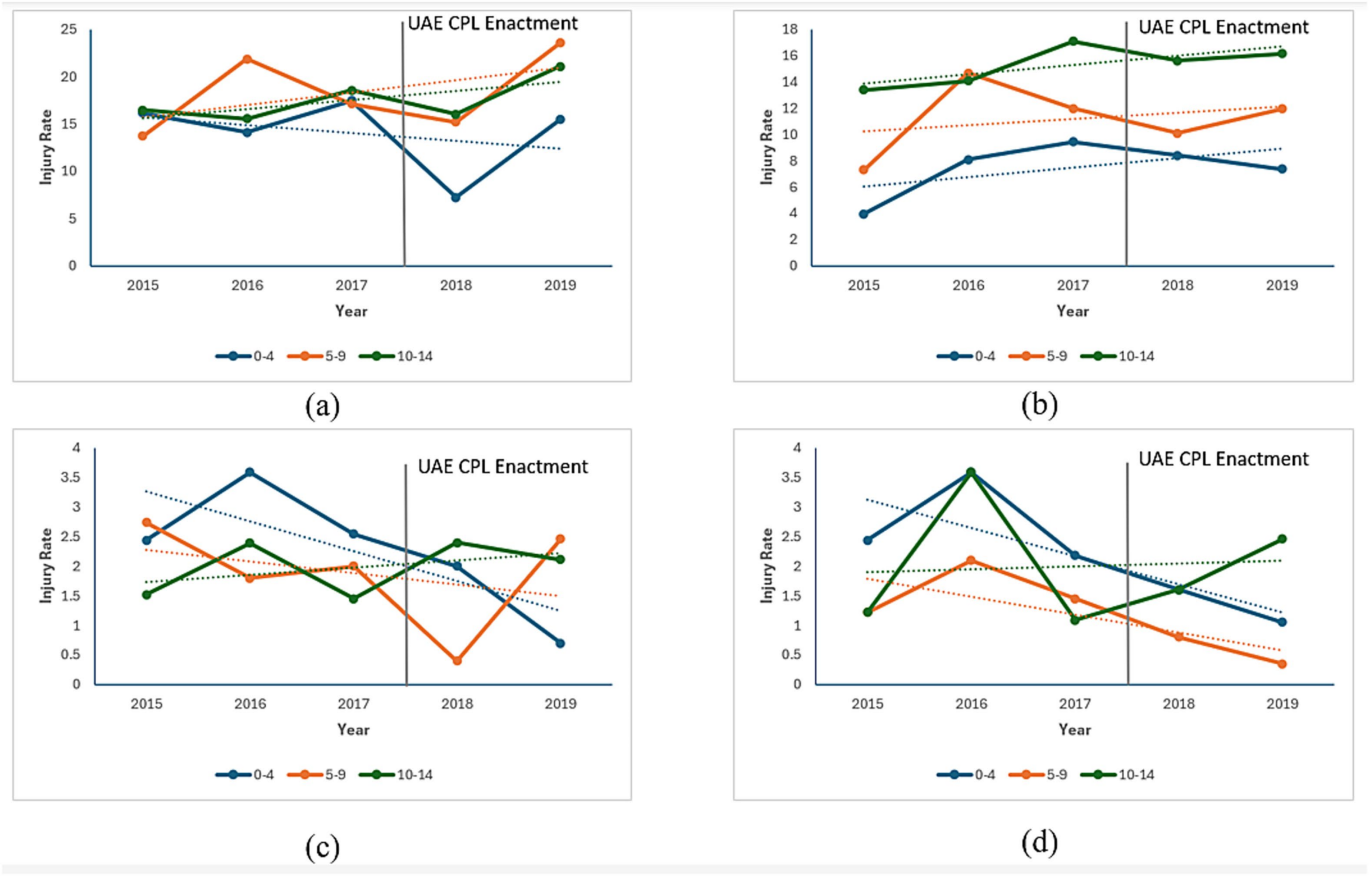
Rate of child occupant injuries **(a)** minor **(b)** moderate **(c)** severe **(d)** fatal.

Figure 1 presents the trend of child occupant injuries between 2015 and 2019. The number of minor injuries sustained by child passengers aged 0–4 years displayed a decreasing trend while there was no change for child occupants aged 5–9 and 10–14 years (Figure 1a). The incidence of moderate injuries did not change over the study period (Figure 1b). Severe (Figure 1c) and fatal (Figure 1d) injuries showed a decreasing trend for child passengers aged 0–4 and 5–9 while no change was observed for child occupants aged 10–14. The rate of minor injuries showed a decreasing trend for child occupants aged 0–4 years while an increasing trend was observed for child passengers aged 5–9 and 10–14 years (Figure 2a). The trend for the rate of moderate injuries for child occupants showed an increasing trend for all the child passengers aged 0–14 years (Figure 2b). Conversely, severe (Figure 2c) and fatal (Figure 2d) injury rates showed a decreasing trend for child passengers aged 0–4 and 5–9 years, while no change was observed for child occupants aged 10–14 years.

**Figure 3:**
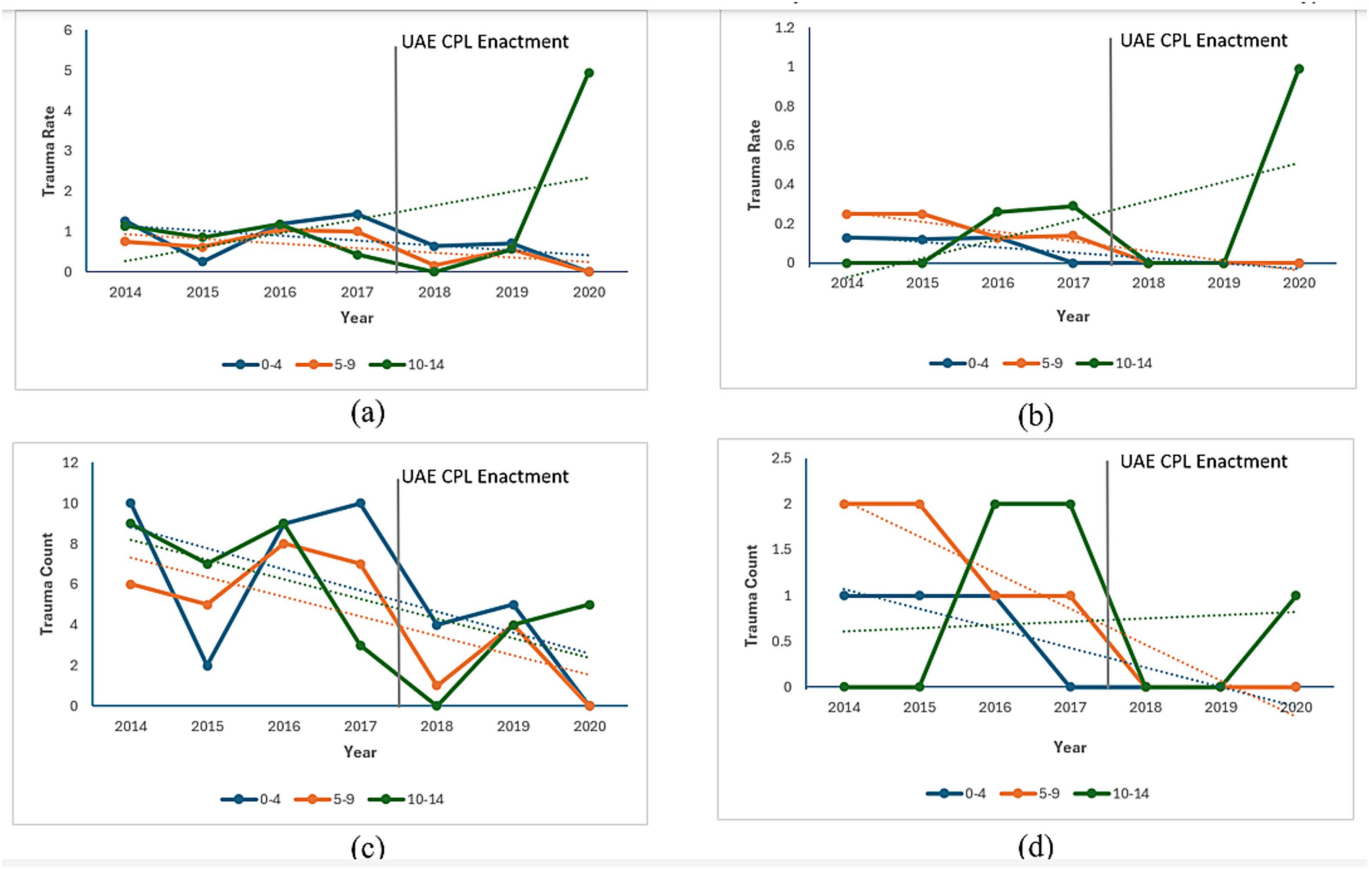
Child occupant trauma **(a)** minor rate **(b)** major rate **(c)** minor count **(d)** major count.

An increasing trend was observed in the rate of minor trauma for child occupants aged 10–14 years, while no change was observed for child passengers aged 0–4 and 5–9 (Figure 3a). The rate of major trauma increased for child occupants aged 10–14 years, while a decreasing trend was observed for child passengers aged 0–4 and 5–9 years (Figure 3b). The incidence of minor trauma displayed a decreasing trend for child occupants aged 0–14 years (Figure 3c). Major trauma showed a decreasing trend for child passengers aged 0–4 and 5–9 years, while no change was observed for child occupants aged 10–14 years during the study period (Figure 3d).

### 3.2 Association between CPL and injury and fatality count reduction

Table 1 presents the results of the association between the CPL and reductions in the absolute counts of injuries and fatalities among children aged 0–4 years. The CPL was significantly associated with a: 32% reduction in the absolute count of minor injuries (IRR: 0.68, CI: 0.51–0.89), 58% reduction in the absolute count of severe injuries (IRR: 0.42, CI: 0.19–0.85), and 68% reduction in the absolute count of fatal injuries (IRR: 0.32, CI: 0.14–0.68). Similarly, the CPL was associated with an 87% reduction in the adjusted count of fatal injuries (IRR: 0.13, CI: 0.01–0.74). The CPL was associated with a significant reduction in the absolute count of fatal injuries (IRR: 0.29, CI: 0.08–0.80) for children aged 5–9 years (Table 2). On the other hand, the CPL was not associated with any significant reduction in injury and fatality counts for children aged 10–14 (Table 3).

**Table 1:** Association between UAE CPL and injury counts for children 0–4 years old.

IRR (95%CI)	Crude model				Adjusted model^*^			
	IRR (95%CI)^1^	IRR (95%CI)^2^	IRR (95%CI)^3^	IRR (95%CI)^4^	IRR (95%CI)^1^	IRR (95%CI)^2^	IRR (95%CI)^3^	IRR (95%CI)^4^
Pre CPL	Ref	Ref	Ref	Ref	Ref	Ref	Ref	Ref
Post CPL	**0.68 (0.51–0.89)**	0.93 (0.64–1.35)	**0.42 (0.19–0.85)**	**0.32 (0.14–0.68)**	1.03 (0.61–1.73)	0.51 (0.23–1.06)	0.44 (0.09–1.70)	**0.13 (0.01–0.74)**

^1^Minor injuries, ^2^moderate injuries, ^3^severe injuries, ^4^fatal injuries. Bold: *p* < 0.05. IRR: incidence rate ratios. CI: confidence interval. ^*^Adjusted for GDP and proportion of young males.

**Table 2:** Association between UAE CPL and injury counts for children 5–9 years old.

IRR (95%CI)	Crude model				Adjusted model^*^			
	IRR (95%CI)^1^	IRR (95%CI)^2^	IRR (95%CI)^3^	IRR (95%CI)^4^	IRR (95%CI)^1^	IRR (95%CI)^2^	IRR (95%CI)^3^	IRR (95%CI)^4^
Pre CPL	Ref	Ref	Ref	Ref	Ref	Ref	Ref	Ref
Post CPL	0.97 (0.76–1.23)	0.79 (0.58–1.08)	0.56 (0.25–1.18)	**0.29 (0.08–0.80)**	1.14 (0.69–1.90)	0.56 (0.29–1.05)	1.04 (0.20–5.35)	0.18 (0.01–1.36)

^1^Minor injuries, ^2^moderate injuries, ^3^severe injuries, ^4^fatal injuries. Bold: *p* < 0.05. IRR: incidence rate ratios. CI: confidence interval. ^*^Adjusted for GDP and proportion of young males.

**Table 3:** Association between UAE CPL and injury counts for children 10–14 years old.

IRR (95%CI)	Crude model				Adjusted model^*^			
	IRR (95%CI)^1^	IRR (95%CI)^2^	IRR (95%CI)^3^	IRR (95%CI)^4^	IRR (95%CI)^1^	IRR (95%CI)^2^	IRR (95%CI)^3^	IRR (95%CI)^4^
Pre CPL	Ref	Ref	Ref	Ref	Ref	Ref	Ref	Ref
Post CPL	1.06 (0.83–1.35)	0.84 (0.65–1.10)	1.07 (0.51–2.24)	0.67 (0.31–1.37)	1.64 (0.99–2.76)	0.60 (0.34–1.02)	1.40 (0.25–8.44)	0.21 (0.01–1.51)

^1^Minor injuries, ^2^moderate injuries, ^3^severe injuries, ^4^fatal injuries. IRR: incidence rate ratios. CI: confidence interval. ^*^Adjusted for GDP and proportion of young males.

### 3.3 Association between CPL and reducing injury and fatality rate reduction

The CPL was significantly associated with a 62 and 85% reduction in the crude (IRR: 0.38, CI: 0.16–0.81) and adjusted (IRR: 0.15, CI: 0.01–0.83) rates of fatal injuries per 1000 crashes for children aged 0–4 years (Table 4). Equally, the CPL was significantly associated with a 66% reduction in the crude rate of fatal injuries (IRR: 0.34, CI: 0.10–0.95) per 1000 crashes for children aged 5–9 years (Table 5). Conversely, the CPL was significantly associated with a 75% increase in the adjusted rate of minor injuries (IRR: 1.75, CI: 1.06–2.95) per 1000 crashes for children aged 10–14 years (Table 6).

**Table 4:** Association between UAE CPL and injury rates per crash for children 0–4 years old.

IRR (95%CI)	Crude model				Adjusted model^*^			
	IRR (95%CI)^1^	IRR (95%CI)^2^	IRR (95%CI)^3^	IRR (95%CI)^4^	IRR (95%CI)^1^	IRR (95%CI)^2^	IRR (95%CI)^3^	IRR (95%CI)^4^
Pre CPL	Ref	Ref	Ref	Ref	Ref	Ref	Ref	Ref
Post CPL	0.81 (0.61–1.06)	1.11 (0.76–1.61)	0.50 (0.23–1.01)	**0.38 (0.16–0.81)**	1.10 (0.65–1.86)	0.54 (0.25–1.11)	0.48 (0.10–1.85)	**0.15 (0.01–0.83)**

^1^Minor injuries, ^2^moderate injuries, ^3^severe injuries, ^4^fatal injuries. Bold: *p* < 0.05. IRR: incidence rate ratios. CI: confidence interval. ^*^Adjusted for GDP and proportion of young males.

**Table 5:** Association between UAE CPL and injury rates per crash for children 5–9 years old.

IRR (95%CI)	Crude model				Adjusted model^*^			
	IRR (95%CI)^1^	IRR (95%CI)^2^	IRR (95%CI)^3^	IRR (95%CI)^4^	IRR (95%CI)^1^	IRR (95%CI)^2^	IRR (95%CI)^3^	IRR (95%CI)^4^
Pre CPL	Ref	Ref	Ref	Ref	Ref	Ref	Ref	Ref
Post CPL	1.16 (0.91–1.47)	0.94 (0.69–1.28)	0.66 (0.29–1.41)	**0.34 (0.10–0.95)**	1.22 (0.74–2.04)	0.60 (0.31–1.11)	1.15 (0.22–5.97)	0.21 (0.01–1.48)

^1^Minor injuries, ^2^moderate injuries, ^3^severe injuries, ^4^fatal injuries. Bold: *p* < 0.05. IRR: incidence rate ratios. CI: confidence interval. ^*^Adjusted for GDP and proportion of young males.

**Table 6:** Association between UAE CPL and injury rates per crash for children 10–14 years old.

IRR (95%CI)	Crude model				Adjusted model^*^			
	IRR (95%CI)^1^	IRR (95%CI)^2^	IRR (95%CI)^3^	IRR (95%CI)^4^	IRR (95%CI)^1^	IRR (95%CI)^2^	IRR (95%CI)^3^	IRR (95%CI)^4^
Pre CPL	Ref	Ref	Ref	Ref	Ref	Ref	Ref	Ref
Post CPL	1.26 (0.98–1.61)	1.00 (0.77–1.31)	1.28 (0.61–2.67)	0.79 (0.37–1.63)	**1.75 (1.06–2.95)**	0.63 (0.36–1.08)	1.51 (0.27–9.26)	0.24 (0.02–1.70)

^1^Minor injuries, ^2^moderate injuries, ^3^severe injuries, ^4^fatal injuries. Bold: *p* < 0.05. IRR: incidence rate ratios. CI: confidence interval. ^*^Adjusted for GDP and proportion of young males.

### 3.4 Association between CPL and trauma count reduction

The CPL was significantly associated with a 99% reduction in the absolute count of major trauma (IRR: 0.01, CI: 0.01–0.85) for children aged 0–4 years (Table 7), 52% reduction in the absolute count of minor trauma (IRR: 0.48, CI: 0.21–0.99) for children aged 5–9 years (Table 8), and 63% reduction in the absolute count of minor trauma (IRR: 0.37, CI: 0.17–0.74) for children aged 10–14 years (Table 9). Likewise, the CPL was associated with a 62% reduction in the adjusted count of minor trauma (IRR: 0.38, CI: 0.17–0.75) for children aged 10–14 years.

**Table 7:** Association between UAE CPL and trauma counts for children 0–4 years old.

IRR (95%CI)	Crude model		Adjusted model^*^	
	IRR (95%CI)^1^	IRR (95%CI)^2^	IRR (95%CI)^1^	IRR (95%CI)^2^
Pre CPL	Ref	Ref	Ref	Ref
Post CPL	0.60 (0.31–1.12)	**0.01 (0.01–0.85)**	0.61 (0.31–1.15)	0.09 (0.01–1.23)

^1^Minor injuries, ^2^major injuries. Bold: *p* < 0.05. IRR: incidence rate ratios. CI: confidence interval. ^*^Adjusted for number of driver license holders.

**Table 8:** Association between UAE CPL and trauma counts for children 5–9 years old.

IRR (95%CI)	Crude model		Adjusted model^*^	
	IRR (95%CI)^1^	IRR (95%CI)^2^	IRR (95%CI)^1^	IRR (95%CI)^2^
Pre CPL	Ref	Ref	Ref	Ref
Post CPL	**0.48 (0.21–0.99)**	0.14 (0.01–2.77)	0.48 (0.22–1.00)	0.18 (0.01–2.36)

^1^Minor injuries, ^2^major injuries. Bold: *p* < 0.05. IRR: incidence rate ratios. CI: confidence interval. ^*^Adjusted for number of driver license holders.

**Table 9:** Association between UAE CPL and trauma counts for children 10–14 years old.

IRR (95%CI)	Crude model		Adjusted model^*^	
	IRR (95%CI)^1^	IRR (95%CI)^2^	IRR (95%CI)^1^	IRR (95%CI)^2^
Pre CPL	Ref	Ref	Ref	Ref
Post CPL	**0.37 (0.17–0.74)**	1.50 (0.25–11.40)	**0.38 (0.17–0.75)**	1.28 (0.19–10.20)

^1^Minor injuries, ^2^major injuries. Bold: *p* < 0.05. IRR: incidence rate ratios. CI: confidence interval. ^*^Adjusted for number of driver license holders.

### 3.5 Association between CPL and reducing trauma rates

The CPL was not associated with any significant reduction in trauma rates per 100 crashes for children aged 0–14 (Supplementary Tables A.3–A.5).

### 3.6 Association between CPL and crash characteristics

Although child passengers in the UAE had 20% lower odds of sustaining more severe injuries in the post-CPL period compared to the pre-CPL period (Table 10), the reduction in the odds of sustaining more severe injuries was not statistically significant (OR: 0.80, CI: 0.34–1.87). Child occupants aged 5–9 years had higher odds of sustaining more severe injuries compared to child occupants aged 0–4 years. Their odds of sustaining severe injuries increased by 108% (OR: 2.08, CI: 1.32–3.27) while their odds of sustaining fatal injuries increased by 177% (OR: 2.77, CI: 1.51–5.10). Similarly, child passengers aged 10–14 years had higher odds of sustaining more severe injuries compared to child passengers aged 0–4 years. Their odds of sustaining severe injuries increased by 155% (OR: 2.55, CI: 1.61–4.04) while their odds of sustaining fatal injuries increased by 175% (OR: 2.75, CI: 1.56–4.86). The odds of sustaining more severe injuries for child occupants decreased by 1% for every 1 unit increase in posted speed (OR: 0.99, CI: 0.98–0.99).

**Table 10:** Relationship between CPL and crash injury characteristics.

Parameter	Cutpoint	Estimate	SE	*p*-value	OR (CI)
Intercept	Constant	0.77	0.31	**0.013**	2.15 (1.18–3.94)
Post CPL	Constant	−0.22	0.43	0.605	0.80 (0.34–1.87)
Age group					
0–4	Ref	Ref	Ref	Ref	Ref
Age group (5–9)	Minor vs. moderate/severe/fatal	0.04	0.18	0.817	1.04 (0.73–1.49)
Age group (5–9)	Minor/moderate vs. severe/fatal	0.73	0.23	**0.001**	2.08 (1.32–3.27)
Age group (5–9)	Minor/moderate/severe vs. fatal	1.02	0.31	**0.001**	2.77 (1.51–5.10)
Age group (10–14)	Minor vs. moderate/severe/fatal	−0.01	0.19	0.993	1.00 (0.69–1.44)
Age group (10–14)	Minor/moderate vs. severe/fatal	0.94	0.23	**<0.001**	2.55 (1.61–4.04)
Age group (10–14)	Minor/moderate/severe vs. fatal	1.01	0.29	**<0.001**	2.75 (1.56–4.86)
Posted speed	Constant	−0.01	0.02	**<0.001**	0.99 (0.98–0.99)
Seating position					
Driver	Ref	Ref	Ref	Ref	Ref
Front	Constant	−0.34	0.19	0.067	0.71 (0.49–1.03)
Rear	Constant	−1.62	0.29	**<0.001**	0.20 (0.11–0.35)
Emirate					
Abu Dhabi	Ref	Ref	Ref	Ref	Ref
Emirate (Sharjah)	Minor vs. moderate/severe/fatal	0.35	0.21	0.094	1.42 (0.94–2.15)
Emirate (Sharjah)	Minor/moderate vs. severe/fatal	−0.49	0.26	0.058	0.61 (0.37–1.02)
Emirate (Sharjah)	Minor/moderate/severe vs. fatal	−0.98	0.31	**0.001**	0.38 (0.20–0.70)
Emirate (RAK)	Minor vs. moderate/severe/fatal	0.48	0.23	**0.034**	1.62 (1.04–2.54)
Emirate (RAK)	Minor/moderate vs. severe/fatal	−0.65	0.26	**0.013**	0.52 (0.31–0.87)
Emirate (RAK)	Minor/moderate/severe vs. fatal	−0.73	0.34	**0.030**	0.48 (0.25–0.93)
Emirate (Fujairah)	Constant	1.37	0.4	**<0.001**	3.93 (1.81–8.55)
Emirate (Others)	Constant	0.37	0.26	0.146	1.45 (0.88–2.41)
Gender					
Girls	Ref	Ref	Ref	Ref	Ref
Boys	Constant	−0.02	0.15	0.90	0.98 (0.73–1.31)
Post CPL × Age group (5–9)	Constant	0.13	0.28	0.63	1.14 (0.67–1.96)
Post CPL × Age group (10–14)	Constant	0.04	0.28	0.99	1.00 (0.58–1.74)
Post CPL × Posted speed	Constant	0.07	0.03	0.06	1.00 (0.99–1.01)
Post CPL × Seating position (front)	Constant	−0.16	0.28	0.58	0.85 (0.49–1.49)
Post CPL × Seating position (rear)	Constant	0.80	0.42	0.05	2.24 (0.99–5.09)
Post CPL × Emirate (Sharjah)	Constant	0.54	0.32	0.09	1.72 (0.92–3.24)
Post CPL × Emirate (RAK)	Constant	−0.34	0.39	0.37	0.71 (0.33–1.51)
Post CPL × Emirate (Fujairah)	Constant	−0.50	0.50	0.31	0.60 (0.23–1.60)
Post CPL × Emirate (others)	Constant	−0.02	0.40	0.97	0.99 (0.45–2.15)
Post CPL × Gender (boys)	Constant	−0.36	0.22	0.11	0.70 (0.45–1.08)

Child passengers seated in the rear seating position had 80% lower odds of sustaining more severe injuries compared with those seated in the driver seating position (OR: 0.20, CI: 0.11–0.35). Child occupants in the emirate of Sharjah had 62% lower odds of sustaining fatal injuries compared to those in Abu Dhabi emirate (OR: 0.38, CI: 0.20–0.70). While child passengers in Ras Al Khaimah emirate had 62% higher odds of sustaining moderate injuries compared to those in Abu Dhabi emirate (OR: 1.62, CI: 1.04–2.54), they had 48 and 52% lower odds of sustaining severe (OR: 0.52, CI: 0.31–0.87) and fatal (OR: 0.48, CI: 0.25–0.93) injuries, respectively, compared to child passengers in Abu Dhabi emirate. On the other hand, child occupants in the emirate of Fujairah had 293% higher odds of sustaining more severe injuries compared to those in Abu Dhabi emirate (OR: 3.93, CI: 1.81–8.55). The interactions between CPL and crash characteristics did not show any significant association with injury severity for child passengers.

There was no significant difference in trauma severity after the implementation of the CPL (OR: 1.64, CI: 0.06–2.72). Compared to child occupants who had a short hospital length of stay (LOS), child occupants who had a long hospital length of stay had significantly higher odds of major trauma (OR: 12.81, CI: 2.28–4.29). While the interaction between CPL and LOS was not statistically significant (Table 11), the estimated effect suggested a reduction in trauma severity (OR: 0.39, CI: 0.01–1.82).

**Table 11:** Relationship between CPL and trauma characteristics.

Variable	Estimate	SE	OR (CI)	*p*-value
Intercept	−3.71	1.01	0.02 (0.01–0.11)	**<0.001**
CPL	0.50	1.44	1.64 (0.06–2.72)	0.731
LOS	2.55	1.08	12.81 (2.28–4.29)	**0.018**
CPL × LOS	−0.94	1.67	0.39 (0.01–1.82)	0.574

AUC: 0.75, classification accuracy: 0.89, *X*^2^(3) = 12.10, *p* = 0.007.

## 4 Discussion

This is the first study to examine the association between the UAE CPL and child occupant injuries and fatalities. The results of our analysis revealed a significant association between the CPL and child occupant injuries and fatalities. The CPL was associated with reductions in the absolute counts of fatal injuries among child occupants aged 0–9 years and in the adjusted counts of fatal injuries among child passengers aged 0–4 years. The crude rate of fatal injuries was also reduced among child occupants aged 0–9 years, but the adjusted rate of fatal injuries was reduced only among child passengers aged 0–4 years. Absolute counts of severe and minor injuries were reduced among child occupants aged 0–4 years. Absolute counts of major trauma were reduced among child passengers aged 0–4 years, while absolute counts of minor trauma were reduced among child occupants aged 5–14 years. However, the adjusted count of minor trauma was reduced only among child passengers aged 10–14 years. These findings suggest that enhanced legislation may be required to achieve comprehensive reductions in child passenger injuries and fatalities.

The study by (17) also found mixed results for the effect of the CPL in Japan on child occupant minor, serious, and fatal injuries. While there was no significant change observed in terms of serious and fatal injuries between the pre- and post-legislation periods, minor injuries were found to have significantly increased post-legislation, which is similar to what was observed in the current study for child occupants aged10-14 years. The authors attributed their findings to the high rates of incorrect CSS use among children in the country, under-enforcement of CSS legislation by law enforcement agencies, and a lack of awareness of correct CSS use.

The introduction of the CPL in Brazil was associated with reductions in hospitalization and death rates post-legislation (18). Similarly, an immediate decrease in child occupant fatalities and a gradual decrease in child occupant injuries over the long term were observed following the implementation of the CPL in Brazil (19). This is somewhat consistent with the results of the present study, which observed significant reductions in child occupant fatalities in the immediate aftermath of the CPL. However, due to the gradual and long-term nature of the effect of CPL on child passenger injuries, the effect of the legislation on minor and moderate injuries was not evident in the current study due to the very short period examined post-legislation (2.5 years). The effects of CPL tend to be gradual due to several factors (20). Hence, a longer post-legislation period is required to assess the effects of the CPL on injury reduction for child occupants (19).

The enactment of CPL in Chile was associated with significant reductions in severe injuries among child passengers (12, 21, 22). A significant decrease in severe injuries to child occupants was also observed in the northern regions of Chile as a result of the CPL, but not at the national level. Additionally, the Chilean CPL was found to result in a significant reduction in child passenger fatalities at the national level as well as in the southern regions (23). In contrast, mixed results were observed regarding the association between CPL in Chile and reductions in child occupant fatalities (12). However, these reductions were short-lived, lasting only 3 years after the legislation took effect. The method employed in the current study does not allow us to evaluate the temporal and territorial effects of the CPL in the UAE, as was performed in the aforementioned study in Chile. Public awareness campaigns about CSS, continued enforcement of the CPL, and education of parents about the safety benefits of restraining their children have been proposed as possible approaches to complement the CPL’s implementation and achieve sustained reductions in child occupant injuries and fatalities (12, 17, 19, 21–23).

As was observed in the current study, several other studies have also reported significant reductions in injuries and fatalities for child occupants after the implementation of CPL. A significant decline was observed in hospital admissions and fatalities in Sweden (24), injuries and fatalities in Israel (25), fatalities in the USA (15), and injuries in Serbia (26). These all point to the effectiveness of CPL in reducing child occupant injuries and fatalities. To the best of our knowledge, this is the first study to examine whether the relationship between crash injury characteristics and injury severity changed after the implementation of the CPL. Our findings indicate no significant associations between crash characteristics and CPL. Also, while the CPL was associated with reducing some of the counts and rates of child passenger injuries and fatalities, especially for child occupants aged 0–4 years, no reductions were observed for child passengers aged 10–14 years.

This may be due to the following reasons: first, the CPL mandates the use of CSS for children without specifying which type of CSS to use from birth until age of 4. This can lead to inappropriate use of CSS with the attendant negative consequences in crash situations. Children are required to travel in rear-facing CSS until age 4 in the Nordic countries (6), whereas in the United States, the recommendation is to use rear-facing CSS until age 2 for better protection (14). It is recommended that children transition to forward-facing CSS only after they have completely outgrown the height and weight limits for rear-facing CSS, typically after age 2 (27). Although vehicle seatbelts have been observed to protect children aged 5 and above compared with travelling completely unrestrained (28), forward-facing CSS or booster seats have been recommended as the best form of restraint for this age group (15).

Based on the findings of this study, the following policy and practice recommendations are proposed:

1 An enhanced CPL is required for better safety outcomes. The new CPL should include the following aspects:

- Mandates the use of rear-facing CSS for children travelling in vehicles until at least age of 2.
- Mandates the use of forward-facing CSS for children travelling in vehicles from ages 2–4 to 8–11.
- Mandates the use of booster seats from age 10 to 14.
- Children should transition from one form of restraint to the next only when they have completely outgrown the weight and height limits of their current restraint for better protection.
- Children should transition to adult seatbelts only when they can achieve appropriate seatbelt fit, or when they reach age 14, according to the weight, height, and seating height requirements for proper seatbelt fit.

2 Strict enforcement of the current as well as the new CPL when it comes to effect by law enforcement officers.
3 Sustained educational and awareness campaigns on the safety benefits of appropriate restraint for children travelling in vehicles.

## 5 Study limitations

This study has some limitations, including an observational study design rather than a causal inference approach, a relatively short post-CPL period analyzed, and the use of data from a single hospital for the trauma severity aspect of the study. Data on CSS use pre- and post-CPL was available only for Abu Dhabi emirate and was missing for all other emirates in the injury severity dataset. Similarly, this data was not recorded for 60% of the cases in the trauma severity dataset. Accordingly, we excluded it from the analyses. Future studies should include longer post-CPL periods and employ causal inference approaches (difference-in-differences, synthetic control, etc.).

## 6 Conclusion

The implementation of UAE’s CPL was associated with significant reductions in injury and fatality counts and rates, especially among children aged 0–4 years. However, more legislative and non-legislative efforts are required for a comprehensive reduction in child passenger injuries and fatalities in the country.

## Data Availability

Publicly available datasets were analyzed in this study. This data can be found at: https://moi.gov.ae/en/open-data/open-data.
