# Supplementary material for: Association between the United Arab Emirates child safety seat and mandatory seatbelt legislation and child passenger injuries and fatalities
Source: Front Public Health. 2026 Jul 20;14:1846288. doi: 10.3389/fpubh.2026.1846288 (PMC13429500; doi:10.3389/fpubh.2026.1846288)
Supplement: Supplementary file 1 [file Table_1.DOCX]

Supplementary Material

# Appendices

Table A.1. Injury severity dataset variables descriptions

| Category | Variable | Values |
| --- | --- | --- |
| Demographic | Gender^†^ | Boy, Girl |
|  | Age^§^ | 0-14 |
|  | Emirate^†^ | Abu Dhabi, Sharjah, Ras Al Khaimah, Fujairah, Others |
| Temporal | Crash time^†^ | Morning, Noon, Afternoon, Evening |
|  | Crash month^†^ | Winter, Summer, Spring, Fall |
|  | Crash day^†^ | Weekdays, Weekends |
|  | Crash week^†^ | First, Second, Third, Fourth, Fifth |
|  | Crash year^§^ |  |
| Environmental | Weather condition^†^ | Clear, Rainy, Others |
|  | Lighting condition^†^ | Day, Night enough lighting, Night no/poor lighting |
|  | Road surface condition^†^ | Dry, Paved, Earthy, Others |
|  | Road condition^†^ | Clear, Curved, Others |
|  | Crash location^†^ | Residential area, Commercial area, Highway, Others |
|  | Intersection type^†^ | None, Roundabout, Semi-vertical, Vertical, Multiple, Others |
|  | Number of lanes^§^ | 1-6 |
|  | Crash site^†^ | Dual carriageway, Highway, Intersection, Single road, Two-way street, Others |
| Crash | Crash type^†^ | Side impact, Rear impact, Rollover, Fixed-object collision, Head-on collision, Perpendicular collision, Others |
|  | Crash cause^†^ | Sudden deviation, Entering main road without checking, Not adhering to mandatory itinerary, Speeding without considering road conditions, Inexperienced/unlicensed driving, Tailgating, jumping red light, Others |
|  | Posted speed limit^§^ | 10-160 |
|  | Seating position^†^ | Driver, Front, Rear, Others |
| Outcome | Injury severity^*^ | Minor, Moderate, Severe, Fatal |
| ^†^Categorical variables  ^§^Numerical variables  ^*^Outcome variable | | |

Table A.2. Trauma severity dataset variables descriptions

| Category | Variable |
| --- | --- |
| Demographic | Age  Gender  Nationality |
| Crash | Crash type  Crash location  Seating position  Restraint status  Vehicle type  Injury type |
| Temporal | Crash month  Crash day  Crash time  Crash week  Crash year |
| Vital signs | Body temperature  Pulse rate  Respiratory rate  Blood pressure  Oxygen saturation  Weight  Height |
| Trauma metrics | Injury severity score  Trauma revised injury severity score  New injury severity score  Revised trauma score  Glasgow coma scale  Maximum abbreviated injury severity score |
| Hospital stay | Number of days in intensive care unit  Number of days requiring ventilation  Emergency department length of stay  Hospital length of stay  Hospital admission unit  Injury details |

Table A.3. Association between CPL and trauma rates per crash for children 0-4 years old

|  | Crude model | | Adjusted model^*^ | |
| --- | --- | --- | --- | --- |
|  | IRR (95%CI)^1^ | IRR (95%CI)^2^ | IRR (95%CI)^1^ | IRR  (95%CI)^2^ |
| Pre CPL | Ref | Ref | Ref | Ref |
| Post CPL | 0.92  (0.47-1.72) | 0.01  (0.01-1.30) | 0.96  (0.49-1.80) | 0.14  (0.01-1.94) |

^1^Minor injuries, ^2^Major injuries. IRR: Incidence rate ratios. CI: Confidence interval.

^*^Adjusted for number of driver license holders.

Table A.4. Association between CPL and trauma rates per crash for children 5-9 years old

|  | Crude model | | Adjusted model^*^ | |
| --- | --- | --- | --- | --- |
|  | IRR (95%CI)^1^ | IRR (95%CI)^2^ | IRR (95%CI)^1^ | IRR  (95%CI)^2^ |
| Pre CPL | Ref | Ref | Ref | Ref |
| Post CPL | 0.73  (0.33-1.51) | 0.22  (0.01-4.24) | 0.76  (0.34-1.57) | 0.27  (0.02-3.69) |

^1^Minor injuries, ^2^Major injuries. IRR: Incidence rate ratios. CI: Confidence interval.

^*^Adjusted for number of driver license holders.

Table A.5. Association between CPL and trauma rates per crash for children 10-14 years old

|  | Crude model | | Adjusted model^*^ | |
| --- | --- | --- | --- | --- |
|  | IRR (95%CI)^1^ | IRR (95%CI)^2^ | IRR (95%CI)^1^ | IRR  (95%CI)^2^ |
| Pre CPL | Ref | Ref | Ref | Ref |
| Post CPL | 0.57  (0.26-1.14) | 2.30  (0.38-17.50) | 0.59  (0.27-1.19) | 1.99  (0.30-15.80) |

^1^Minor injuries, ^2^Major injuries. IRR: Incidence rate ratios. CI: Confidence interval.

^*^Adjusted for number of driver license holders.
